# Intrinsic MRI visualizes RF lesions within minutes after MR-guided ablation

**DOI:** 10.1186/1532-429X-18-S1-P206

**Published:** 2016-01-27

**Authors:** Philippa Krahn, Venkat Ramanan, Labonny Biswas, Robert Xu, Jennifer Barry, Nicolas Yak, Kevan Anderson, Sheldon Singh, Mihaela Pop, Graham A Wright

**Affiliations:** 1 Physical Sciences Platform, Sunnybrook Research Institute, Toronto, ON Canada; 2 Medical Biophysics, University of Toronto, Toronto, ON Canada; 3 Cardiology, Sunnybrook Health Sciences Centre, Toronto, ON Canada

## Background

MR visualization of RF lesions is an application of growing interest with the potential for translation to clinical ablation procedures. In particular, intrinsic-contrast MRI avoids the dynamic contrast produced in typical Gd-based MRI, and may differentiate the reversible and irreversible thermal injury thought to be caused by RF ablation. This distinction is important for assessing the permanence of ablation to eradicate the substrate of ventricular tachycardia in structural heart disease. In this study we investigate the potential of intrinsic-contrast MRI to visualize the features of thermal injury and evolution of RF lesions that may occur immediately after ablation.

## Methods

8 RF ablation lesions were created *in vivo* in 4 healthy pigs. Active real-time MR tracking [1] guided navigation of an MR-enabled catheter (Imricor Medical Systems) within the LV. During ablation, 30-40W was applied to the LV endocardium for 45-60s with catheter tip irrigation throughout. MR images were acquired repeatedly during the ensuing 1-2h, a time frame relevant to the length of clinical ablation procedures. The imaging protocol consisted primarily of T_2_-prepared b-SSFP for T_2_ mapping and IR-prepared b-SSFP. In T_2_ maps, long-T_2_ regions representative of inflamed, edematous tissue were delineated semi-automatically using a threshold of T_2_=55ms, approximately 3SD above remote as per established methods describing edema in T_2_-weighted images [2]. Further, we manually delineated the core of RF lesions based on myocardial hyperenhancement in IR-SSFP images as in [3]. In all analysis, one-tailed t-test was used and p < 0.05 considered significant.

## Results

**Figure 1:**
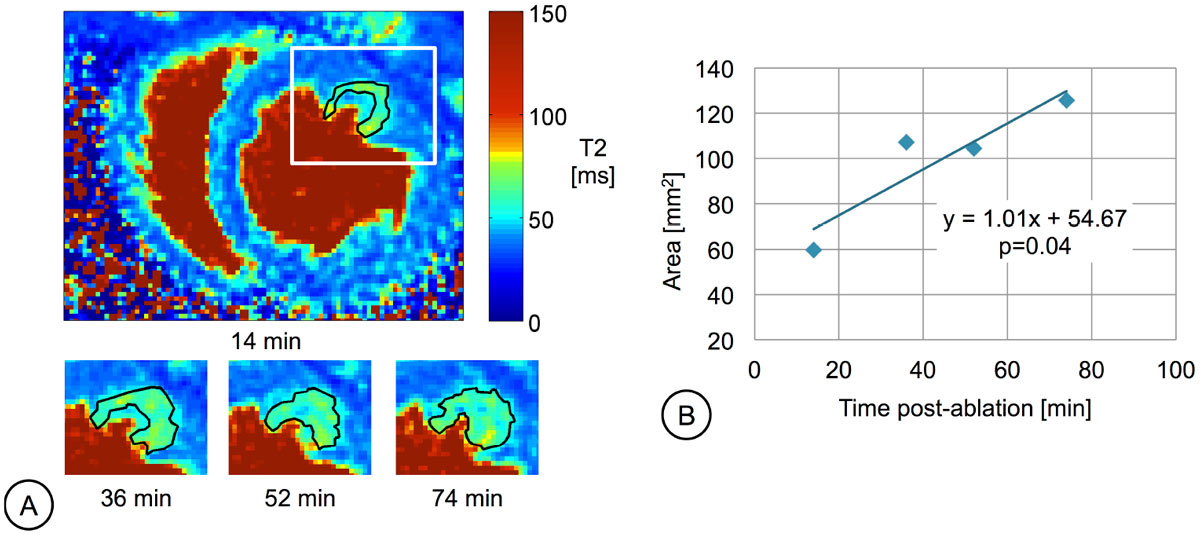
**(a) T**_**2**_ **maps depicting an RF lesion at 4 time points after ablation**. Images were acquired using a 4-channel cardiac anterior array at 4 echo times: TE=3, 24, 88, & 184 ms and fitted using a 3-parameter model (resolution = 0.9 × 0.9 × 6 mm). **(b)** Area of edema with time after ablation for the same lesion. The edematous region grew in size by 110%, and this positive trend was significant with p < 0.05 for the fitted line.

**Figure 2:**
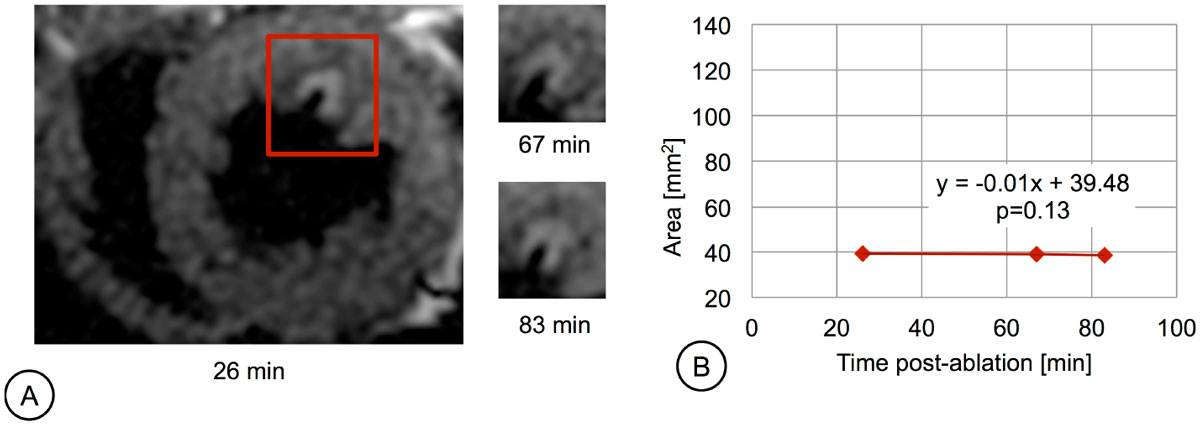
**(a) IR-SSFP images depicting an RF lesion (same lesion as shown in Figure 1) at 3 time points after ablation**. Images were acquired using the following parameters: TE/TR=2/6 ms, resolution = 0.9 × 0.9 × 6 mm, and TI=730-775 ms (within the range of TI yielding optimal contrast [3]). **(b)** Area of the necrotic core with time after ablation for the same lesion. The slope of the fitted line was not significantly different from 0.

In a subset of 4 lesions, the earliest T_2_-prep acquisition was 9 min post-ablation and clearly demonstrated edema at the ablation site. Overall, T_2_ in the edematous and remote tissue was 64.8 ms and 40.7 ms respectively. The edematous areas increased markedly with time post-ablation, by 89% on average. This trend was significant for the exemplary lesion in Figure 1, where p < 0.05 (rejecting the null hypothesis that the slope is 0), and edema size seemed to stabilize after 36 min. In a second subset of 4 lesions, the earliest IR-SSFP acquisition was 3 min post-ablation and these images clearly demonstrated the lesion core. The exemplary lesion core in Figure 2 remained stable over time (p>0.05 indicating no change in the core area). Although limited by noise in initial images acquired with a surface coil, preliminary results support the stabilization of lesion core.

## Conclusions

We successfully demonstrated the visualization of RF lesions using intrinsic-contrast MRI during a time frame spanning minutes to hours after ablation. The presence of edema is of particular interest as it is thought to temporarily alter myocardial excitability, confounding clinical tests used to confirm RF ablation procedural success. This valuable description of RF lesions could be integral in future ablation procedures performed concurrently with MRI feedback.

